# The global forum on bioethics in research meeting, “ethics of research in pregnancy”: emerging consensus themes and outputs

**DOI:** 10.1186/s12978-017-0431-1

**Published:** 2017-12-14

**Authors:** Adrienne Hunt, Natalie Banner, Katherine Littler

**Affiliations:** Policy Department, The Wellcome Trust, London, UK

**Keywords:** International research ethics, Global forum on bioethics in research, Pregnancy

## Abstract

Research during pregnancy is affected by multiple ethical challenges which have not received sufficient international attention and consideration from the bioethics, clinical, and policymaking communities working together. Unresolved ethical questions about research in pregnancy have significant detrimental impacts on maternal and newborn health, in part because they inhibit an evidence base being developed on the efficacy and safety of medicines and health interventions for pregnant women. These problems are compounded in low- and middle-income country (LMIC) settings due to variability in regulatory provisions, the burden of maternal morbidity and mortality, and many social and cultural conventions that impact on pregnant women’s ability to participate in research. Research in pregnancy was chosen as a topic for the 2016 Global Forum on Bioethics in Research (GFBR) meeting, and its timeliness was all the more apparent given the 2016 Zika outbreak, which has deeply affected the Latin American region. The meeting’s emerging consensus themes and outputs epitomized the core aims of the GFBR—to give voice to LMIC perspectives as a priority in dialogue about global health research ethics and to promote collaboration. In this instance, the GFBR meeting catalyzed a strong, unified drive to push researchers and policymakers to include pregnant women in research by default: given the complex nature of the topic, this is a significant achievement in addressing an important question of social justice.

## Background

Ethical controversies about research can be detrimental for global health. Promising research and innovations can be delayed, undermined, or disadvantage the communities they wish to serve because there has been a failure to recognize the depth and importance of the ethical issues associated with that research. For example, a lack of community engagement about research can lead to significant distrust and reluctance to participate in vaccine trials, as was evident during the Ebola outbreak [1]. Despite the existence of international guidelines on many aspects of ethical research, these are not always sufficient in themselves; their effectiveness depends on the systems in which they are implemented. Questions about the appropriateness and sensitivity of research and guidelines to the local context are often most acute for research conducted in low- and middle-income countries (LMICs) and have resulted in a long history of concerns about exploitation and unethical practice [2].

The Global Forum on Bioethics in Research (GFBR) was created in the late 1990s by a group of global health research funders as a way of addressing this concern, seeking to amplify the voices of LMIC partners and facilitate better relationships with them [3]. The GFBR holds an annual meeting centered on a key emerging theme of significance for global health research. Pregnancy is a critical focus for global health research that has not received sufficient international attention and consideration from the bioethics, clinical and policy-making communities working together. Unresolved ethical questions about research in pregnancy have significant detrimental impacts on maternal and newborn health, in part because they inhibit an evidence base being developed on the efficacy and safety of medicines and health interventions for pregnant women. These problems are compounded in LMIC settings due to variability in regulatory provisions, the burden of maternal morbidity and mortality, and many social and cultural conventions that impact on pregnant women’s ability to participate in research.

**Fig. 1:**
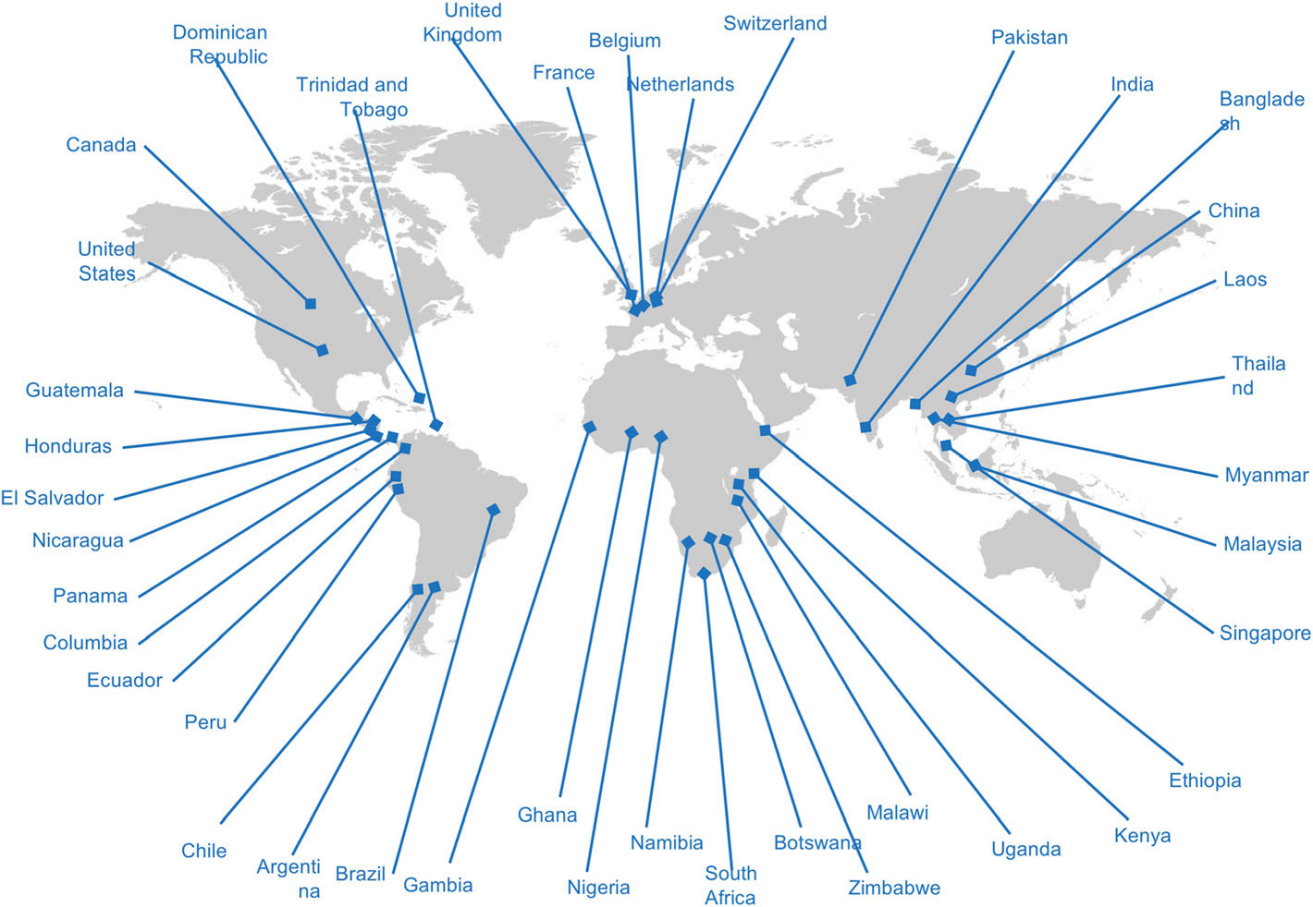
GFBR participants 96 participants from 40 countries came together to discuss this important issue with a wide range of academic and clinical expertise: bioethicists, clinicians, community practitioners, policymakers, social scientists, regulators, and funders, at all levels of seniority. 61 participants were from LMICs

Research in pregnancy was therefore chosen as the topic for the 2016 GFBR meeting and its timeliness was all the more apparent given the 2016 Zika outbreak, which has deeply affected the Latin American region and highlighted the importance of including pregnant women in research [4]. The meeting was held in Buenos Aires, Argentina, over two days and brought together stakeholders from 40 countries (Fig. 1), across fields of bioethics, epidemiology, law, medicine, nursing, policymaking, and industry, to consider the ethical challenges for research in pregnancy that span cultures and geography. Using a case study format that enabled participants to understand the practical issues “on the ground” in addition to broader ethical and policy questions (Table 1), the meeting revealed complex issues and a diversity of experiences [5].

**Table 1:** GFBR meeting format

The case studies contained in this Supplement Issue formed the basis of the GFBR meeting and were themed by context: pregnancy specific research, non-communicable disease, communicable disease, and public health emergencies. Each session concluded with plenary discussion and was followed by intense, small group discussion. The small groups comprised geographically diverse participants so each could learn from the others’ experience and point of view. A dedicated session on policy and regulation gave four perspectives on the issue of research in pregnancy: a global view, focusing on the Council of International Organizations of Medical Sciences guidelines on research with pregnant and breastfeeding women, a view through the lens of US regulation, a regional view from Latin America, and a local perspective drawing on the experience of conducting research on the Thai-Burmese border.	

### Emerging consensus themes

#### Pregnant women should not be excluded from research by default

Despite the breadth of perspectives presented, a strong cross-cultural consensus emerged that the policy of ‘exclusion by default’ needs to change and that pregnant women should be included in research unless there are valid reasons to specifically exclude them. Pregnant women are typically excluded from research because they are categorized as “vulnerable”, which has led to less research being conducted with pregnant women. This ironically results in the population labelled as “vulnerable” actually being put at risk of potentially harmful clinical interventions, under-treatment, or failed prevention of maternal (and sometimes fetal) disease because we lack a solid evidence base. GFBR participants agreed that alternative categories to the traditional classification as “vulnerable” are required: pregnant women are not vulnerable in any cognitive or physical sense, but they are a special research population since pregnancy does pose scientific challenges and specific circumstances, such as risk of harm to the fetus, which may require special protections. This means that there is no justification for excluding pregnant women from research protocols by default.

#### Assessment of risks should include the risks of a pregnant woman *not* participating in research

Decisions about inclusion should be based on principles of justice, the need for evidence on efficacy and balanced assessments of maternal and foetal risk. Participants agreed that as protocols are developed, consideration should be given not only to the risks of participation but also to the risks to the pregnant woman and/or fetus if she is *not* included in the research. Research ethics committees (RECs) should be encouraged to consider both the risks of participation and non-participation for the woman and fetus, bearing in mind it is in the interests of a fetus to have a healthy mother. Researchers should be required to justify why pregnant women should be excluded from research if there is a possibility that the research may benefit the pregnant woman, the fetus, or the future child individually, or may benefit pregnant women or the children they will bear as a class.

#### Cultural norms for consent should be taken into account

A recurring theme throughout the meeting was that cultural views can pose a significant barrier to the participation of pregnant women in research. For example, should consent be sought only from the women as individual research participants or should it be widened to their family network? Even where national law and international guidance only recognize individual consent, in practice, family decision-making or agreement may be the culturally favoured approach, especially when women are pregnant. Since in many settings this is the cultural norm, GFBR participants considered it acceptable to integrate the consultation and engagement of other relevant family members in the consent and enrolment process, providing the final consent is given by the pregnant woman.

#### Community engagement is vital for addressing cultural norms or beliefs that may lead to exclusion of pregnant women

Other cultural barriers were identified including differing views about the value, appropriateness, or safety of study interventions: one case study highlighted that the difficulties of recruiting pregnant women into research on lifestyle or nutrition interventions to prevent gestational diabetes, on the belief that mothers-to-be should lead a sedentary lifestyle and consume high-calorie food. In another case, a study’s procedures to take blood samples gave rise to concerns and refusal from some pregnant women and their families, on the belief that any loss of blood would harm the mother and the fetus. GFBR participants agreed that this speaks to the broader need for robust engagement strategies to reconcile cultural norms and beliefs with the ethical and clinical rationale supporting the need for research during pregnancy. Such engagement should include the pregnant women, their families and community, healthcare providers, RECs, and research funders.

#### Research protocols and agreements with pharmaceutical companies, government, and research funders should be drafted in inter-epidemic periods to ensure there is adequate consideration of and planning for how to include pregnant women in public health emergency research

Public health emergencies present particular challenges for the inclusion of pregnant women given the need for a rapid response and use of experimental treatments, which may not have regulatory approval for use during pregnancy. Pharmaceutical companies often wish to exclude pregnant women from protocols in these situations because of legal concerns. Discussion gave rise to a number of practical recommendations including the need to develop international guidelines for emergency research with pregnant women during epidemics based on the experience in past epidemics. Study files with templates for emergency research consent should also be developed so they are in place prior to future epidemics, and trial insurance should be adapted to emergency situations to reduce liability risks for drug manufacturers so that they will permit pregnant women to be included in emergency research. Drug and vaccine manufacturers need to be more actively engaged in an ongoing dialogue to enable appropriate research and development that better fits the needs of pregnant women.

### Meeting outputs

In selecting GFBR participants, the intention is to consider both potential to actively contribute to the discussions and to achieve impact after the meeting. Participants are encouraged to report the meeting recommendations in their home countries and to continue the discussion in their local context. GFBR participants have given presentations on the ethics of research in pregnancy to their local RECs and at other conferences. For example, the topic was presented at the Medical Research Council of Zimbabwe’s Annual Health Research Forum 2016 while a Zika case study was translated into Spanish and adapted for presentation at the annual workshop of the Network of Human Research Ethics Committees of Cali, Colombia – a country significantly affected by the Zika outbreak.

One of the most significant outputs to date came from a group of Latin American participants, including ethicists, researchers, ethics committee members and representatives of health authorities from Argentina, Brazil, Chile, Colombia, Ecuador, El Salvador, Guatemala, Honduras, Panama, Peru, Nicaragua and Dominican Republic. The group published a consensus statement in the Pan American Health Organization’s Public Health Journal, which is widely read in the region and thus highly influential. At a time when many Latin American countries are reviewing their regulatory frameworks for research with human subjects, the authors call for action and the ‘responsible inclusion of pregnant women in research [as] a matter of equity and social justice’ [6]. This output exemplifies two of the key aims of the GFBR: to develop and promote the highest standards in ethical practice globally, and to enable new networks and collaborations to form across disciplines and countries in order to address challenges from the context where research is being conducted and is most needed.

### Fellowship scheme

The GFBR fellowship scheme presents a unique opportunity for those attending the forum to apply to work in partnership with other attendees to further explore and address the ethical challenges that are identified during the GFBR meeting [7]. The scheme is a further way in which the GFBR seeks to promote practical outputs and discussion beyond its annual meeting.

After the 2016 meeting, nine fellowships were awarded by a competitive process to build upon ideas and discussions about improving ethical practice for research in pregnancy. The GFBR Fellows come from Botswana, Zimbabwe, Malawi, Kenya, China, Honduras, Brazil, and Argentina, and their collaborators are drawn from South Africa, the Gambia, Singapore, Thailand, Netherlands, United Kingdom, Dominican Republic, Canada, the United States, and Peru. The Fellows will create open educational resources on research in pregnancy for RECs and others, develop reference documents to incorporate pregnant women in vaccine clinical development plans (e.g., assessing alternative trial designs and trials for specific gestation periods), explore the perceptions of key stakeholders regarding the complexities of conducting research with pregnant women affected by HIV, and review reasons for exclusion in malaria drug clinical trials.

The fellowships not only promote new collaborations but ensure the legacy of the 2016 GFBR meeting by giving rise to outputs that further encourage dissemination and discussion about this vitally important area of global maternal, fetal, and neonatal health.

The meeting’s emerging consensus themes and outputs epitomized the core aim of the GFBR, to give voice to LMIC perspectives as a priority in dialogue about global health research ethics. In this instance, the GFBR meeting catalyzed a strong, unified drive to push researchers and policy-makers to include pregnant women in research by default: given the complex nature of the topic, this is a significant achievement in addressing an important question of social justice.

## Abbreviations

GFBR: Global Forum on Bioethics in Research
LMIC: Low- and middle-income country
REC: Research ethics committee
